# Analytical Modeling of the Interaction of a Four Implant-Supported Overdenture with Bone Tissue

**DOI:** 10.3390/ma15072398

**Published:** 2022-03-24

**Authors:** Bohdan Pelekhan, Maciej Dutkiewicz, Ivan Shatskyi, Andrii Velychkovych, Mykola Rozhko, Liubomyr Pelekhan

**Affiliations:** 1 Department of Dentistry of Postgraduate Study Faculty, Ivano-Frankivsk National Medical University, Halytska Str. 2, 76018 Ivano-Frankivsk, Ukraine; bpelechan@gmail.com (B.P.); rector@ifnmu.edu.ua (M.R.); pelekhan.liubomir@gmail.com (L.P.); 2 Faculty of Civil and Environmental Engineering and Architecture, Bydgoszcz University of Science and Technology, Kaliskiego 7, 85-796 Bydgoszcz, Poland; 3 Laboratory of Modeling of Damping Systems, Pidstryhach-Institute for Applied Problems in Mechanics and Mathematics of the National Academy of Sciences of Ukraine, Mykytynetska Str. 3, 76002 Ivano-Frankivsk, Ukraine; ipshatsky@gmail.com; 4 Department of Construction and Civil Engineering, Ivano-Frankivsk National Technical University of Oil and Gas, Karpatska Str. 15, 76019 Ivano-Frankivsk, Ukraine; a_velychkovych@ukr.net

**Keywords:** stress state, contact interaction, bar fixation system, overdenture, dental implant, distal cantilever

## Abstract

Today, an interdisciplinary approach to solving the problems of implantology is key to the effective use of intraosseous dental implantations. The functional properties of restoration structures for the dentition depend significantly on the mechanical stresses that occur in the structural elements and bone tissues in response to mastication loads. An orthopedic design with a bar fixation system connected to implants may be considered to restore an edentulous mandible using an overdenture. In this study, the problem of the mechanics of a complete overdenture based on a bar and four implants was formulated. A mathematical model of the interaction between the orthopedic structure and jawbone was developed, and a methodology was established for the analytical study of the stress state of the implants and adjacent bone tissue under the action of a chewing load. The novelty of the proposed model is that it operates with the minimum possible set of input data and provides adequate estimates of the most significant output parameters that are necessary for practical application. The obtained analytical results are illustrated by two examples of calculating the equivalent stresses in implants and the peri-implant tissue for real overdenture designs. To carry out the final assessment of the strength of the implants and bone, the prosthesis was loaded with mastication loads of different localization. In particular, the possibilities of loading the prosthesis in the area of the sixth and seventh teeth were investigated. Recommendations on the configuration of the distal cantilever of the overdenture and the acceptable level and distribution of the mastication load are presented. It was determined that, from a mechanical point of view, the considered orthopedic systems are capable of providing long-term success if they are used in accordance with established restrictions and recommendations.

## 1. Introduction

The restoration of integrity in edentulous areas using intraosseous dental implantation allows the practitioner to achieve clearly predictable results in the complex functional and aesthetic rehabilitation of the patient.

The effectiveness of orthopedic prostheses based on intraosseous dental implants is higher than that of “traditional” orthopedic prostheses. The technique of dental implantation is advantageous since it allows for the individualization of the patient’s orthopedic treatment by making both removable and fixed overdentures that have more natural biomechanics, allowing the practitioner to save the patient’s own teeth [1]. The main advantage of implant-supported overdentures is their method of distribution of the occlusal load: it is more physiologically (homogeneously) distributed in the bone tissue of the jaw without overloading the surrounding teeth and without adverse effects on the oral mucosa [2]. However, adequate qualitative and quantitative indicators of bone tissue are required for the placement and sufficient primary stabilization of dental implants [3].

Currently, the most successful paradigm in implant treatment planning is a multidisciplinary approach. Before surgery, based on the anatomical features of the jaws and occlusal planes, the dentist and the orthopedist draw up a treatment plan and determine the required number and placement of dental implants [4].

It is known that with careful adherence to the protocol (technology) of dental implantation, it is usually possible to achieve implant osseointegration; however, along with this, one should be sure that the integrated implants and bone tissue can withstand the occlusal load transferred to them during masticatory function. When using dental prostheses designed to treat edentulism, cross-stabilization is created; in this case, the force vectors differ significantly from such vectors when using dental bridges, where there is no stabilization. If there is a distal cantilever or a separate implant, lateral loading must be considered. The more attention paid to such issues, the higher the effectiveness of the implant treatment [5]. At first, it was believed that the functioning of intraosseous dental implants was achieved using any type of dental prostheses. This, perhaps, limited the period of effectiveness of implants that was reached—5–7 years. Today, the principles of treatment planning as well as the methods used during the surgical stage of treatment have changed, and methods for determining and analyzing occlusion at the stage of fixation and its control in remote periods of time are being developed [6,7,8]. All this is important to increase the effectiveness of the functional and aesthetic rehabilitation of patients using the dental implantation technique.

It is recognized that the implant and bone tissue must be subjected to a limited range of stress in order to maintain physiological homeostasis. Overloading can cause bone resorption or a fatiguing fracture at the implant neck, while underloading can cause atrophy and bone loss [9].

From the point of view of biomechanics, the successful functioning of dental implants depends on how mechanical loads are transmitted to the implant and peri-implant tissues and what values of stresses arise [10]. Multiple factors influence the occurrence of deformations and stresses, including the place of application, the direction and magnitude of the load, the number and location of the implants, the length and diameter of the implant, the geometry of the implant and its surface structure, the quality of the environment and bones, etc. Only by understanding which of these factors are significant and which are secondary is it possible to develop the right orthopedic treatment strategies based on dental implants.

The effectiveness of implant treatment protocols has been confirmed by evidence-based medicine. In the edentulous mandible, an effective method of restoring the dentition is the manufacture of a bar supported by dental implants and a completely removable overdenture supported by a fixed bar [11,12,13,14].

In general, an implant-supported bar retaining overdenture system can be used to restore maxillary or mandibular edentulousness with a removable overdenture. In one study, a great deal of evidence for the successful functioning of this type of orthopedic prosthesis was reported with its early assembly for 48 h to 3 months from the moment of placement of four implants in the mandibular dentition or six implants in the maxilla [15]. Bar retaining overdentures have a number of advantages, for example, the removable overdenture can be reliably fixed, the patient can easily remove and install the overdenture on the bar, the hygienic care of the prosthesis and bar is simple; the conditionally removable bar can be removed and replaced with a new one, the installed overdenture does not move while eating, and thus does not cause discomfort and inconvenience [16,17]. Usually, an orthopedic structure with a bar retaining system is placed in several clinical and laboratory stages [18]: After planning the orthopedic treatment, implants are placed. Based on an impression taken using the open tray method, a model is made with analogues of the implants, on which the central ratio of the jaws and height are fixed. A bar is modeled digitally and then cast; this will splint the implants and act as a retainer for a complete removable overdenture. After fitting the bar, an individual cover prosthesis is made according to the impression and the structure is fixed. Providing the patient with recommendations for oral care [19] and rules for the removal and insertion of the overdenture is important for maintaining its function.

The choice of dental implant configuration depends on a number of criteria, among which the development of the future orthopedic structure and the anatomical features of the patient are the most important. Biomechanical criteria are of paramount importance and should be developed to increase the likelihood of implantation success, especially in difficult clinical cases [20,21]. From the point of view of mechanics, the problem with the interaction between an implant and the bone tissue is a contact problem of the interaction of two bodies with different physical and mechanical characteristics. In general, contact problems for elastic systems constitute an actual section of the mechanics of a deformable solid body. Statements and methods for solving contact problems using continuum models of a continuous medium have been developed [22,23,24]. The results described in such papers reveal the general regularities of the contact interaction of rigid bodies. Here, we will briefly note the main approaches related to the problem considered in this article. A number of modern studies are devoted to contact problems for shell rod systems, taking into account the energy dissipation during mutual slippage in contact pairs [25,26,27]. Analytical [28,29,30], analytic-numerical [31,32,33], and experimental [34,35] approaches to the study of stresses in composite, substantially inhomogeneous structures also deserve attention.

Since the rigidity of the implant is much greater than the rigidity of the bone tissue, the implant in the bone can be considered as an absolutely rigid linear inclusion interacting with an elastic body. Models and analytical methods for studying the behavior of thin inclusions in elastic media are considered in several monographs [36,37] and articles [38,39,40]. One publication [41], which proposed an approach to the analysis of stresses in an array reinforced with an ensemble of rigid inclusions connected to a single framework, deserves special attention. Separate problems regarding the inelastic interaction of a rigid linear inclusion with a matrix were considered in [42,43].

Winkler’s hypothesis about the proportionality of stresses and discontinuities on the model interface [22,23,24] is very fruitful in problems considering the conjugation of continuums through a thin intermediate layer, and makes it possible to reduce the dimension of the problem. Examples of the successful application of this model are found in [44,45,46,47] for one-dimensional rod objects and [48,49,50] for two-dimensional structures. In particular, in dentistry, the Winkler layer can describe the behavior of periodontal tissues in the interaction of the dental root with the jaw [51].

The cantilever effect caused by the localization of the mastication load on the molars in the prosthetic version leads to a significantly off-center load on the implants. Some approaches to the problem of centering linear objects are considered in [52,53,54,55]. The issues surrounding the strength of plate structures under conditions of simultaneous tension (compression) and bending, taking into account the closure of crack-like defects, were studied in [56,57,58]; similar problems for cracked shallow shells were considered in [59]. In this case, the model of partial contact along the line turned out to be quite effective [60,61,62].

The functional properties of the restoration structure of the dentition depend significantly on the mechanical stresses that occur in the structural elements and bone tissues in response to mastication loads. Occlusal overload is one of the main causes of failure in dental implant treatment [63,64,65]. Therefore, to select an adequate method for tooth restoration and the parametric optimization of the restoration structure, a careful calculation of the indicators of its stress–strain state is required. The insufficient study of this issue by analytical means and the inconvenience of the practical application of the existing results were the main motivation for our study.

The aim of this work was to build analytical estimates of the stress state of implants and the adjacent bone tissue, as well as to develop recommendations on the acceptable level and distribution of the mastication load. To do this, the main tasks outlined in the article, organized in this way, are solved. First, the problem of the mechanics of a complete four implant-supported overdenture is formulated; then, a mathematical model of the interaction of a rigid frame with the jawbone is developed, and the methodology for an analytical study of the stress state of implants and their adjacent bone tissue under a mastication load is described. The analytical results obtained in the next section are illustrated by two examples of the calculation of equivalent stresses in real prosthesis designs. We also provide some guidelines for distal cantilever configuration. A brief discussion and conclusions conclude the publication.

## 2. Materials and Methods

### 2.1. Formulation of the Problem of the Mechanics of a Four Implant-Supported Overdenture

**Figure 1:**
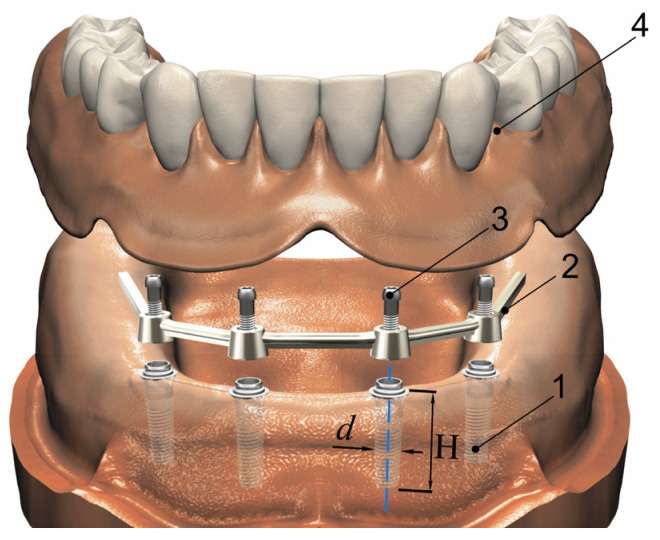
Four implant-retained mandibular bar overdenture scheme: 1—implant; 2—bar; 3—screw; 4—removable overdenture; *H* and *d* are the length and the diameter of implant.

The design of the orthopedic structure based on four implants is shown in Figure 1. Four intraosseous implants were placed in the edentulous mandible. A metal bar was mounted on them, combining the implants into a single orthopedic structure. On the bar, through a system of attachments or directly with slight tension, a complete removable overdenture was fixed—an imitator of the dental arch.

**Figure 2:**
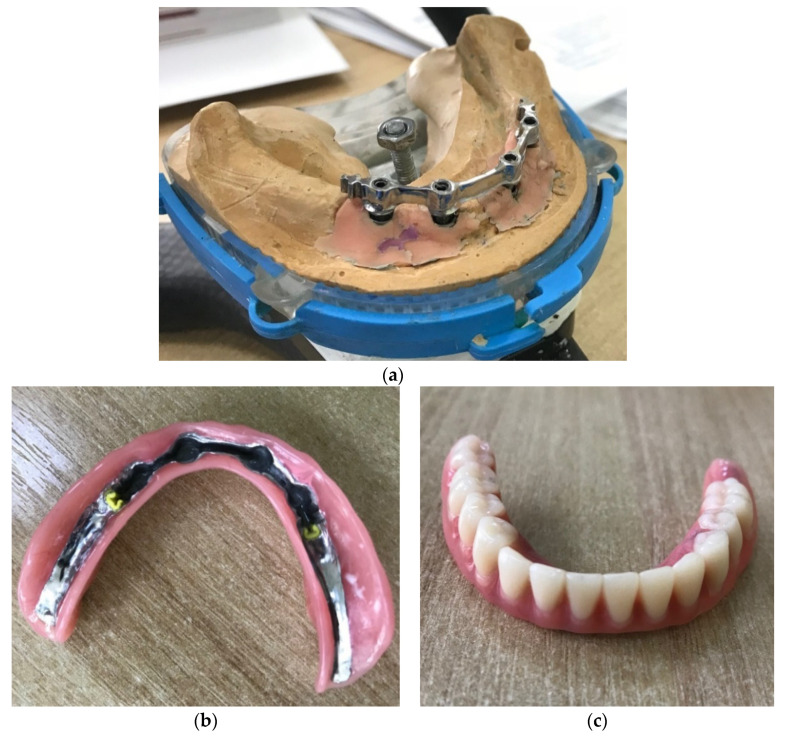
Individual components of the bar structure during the manufacturing process: (**a**) bar, (**b**) secondary overdenture frame, and (**c**) removable overdenture.

Note that the removable overdenture (4) is fixed rigidly to the bar with the help of a metal socket (Figure 2). Due to the fact that the spatial geometry of the secondary frame in the overdenture (Figure 2b) corresponds to the bar geometry (Figure 2a), forming a so-called lock when connected, the overdenture is securely fixed in the oral cavity. Mastication forces are transmitted through the artificial teeth of the overdenture to the metal prosthetic socket and further to the supporting elements (bar and implants).

Therefore, the overdenture is placed under a given vertical mastication load. The distribution of stresses and displacements in the implants and the surrounding bone must be assessed.

### 2.2. Mathematical Model of the Interaction of a Four-Implant Overdenture with Bone Tissue Research Methodology

#### 2.2.1. Basic Assumptions of the Model

In order to obtain results in an analytical form, we accepted the following main hypotheses:

(1) The metal structure, including 4 implants, a unifying polygonal bar, and an arched base of the dentition, is a solid and absolutely rigid spatial frame;
(2) The bone in the vicinity of the implant is modeled by a homogeneous elastic Winkler layer [22,51,66];
(3) The mechanical contact between the components is considered perfect.

The first assumption is a consequence of the fact that the modulus of elasticity of the metal frame is two orders of magnitude higher than the modulus of elasticity of the bone tissue. In addition, from the point of view of kinematics, a solid and absolutely rigid object has a limited number of degrees of freedom.

The second assumption allowed us to use the simplest traditional model of the contact interaction of a solid body (implant) with a deformable body (bone). To simplify the analysis, we also considered the bone to be uniform in height with an average modulus of elasticity of the cortical and trabecular layers.

Finally, the third assumption meant that we did not take into account the phenomenon of bone resorption at areas in contact with the implant.

#### 2.2.2. Coordinate Systems and Some Vector Operations

Without a loss of generality, we assume that the length of all implants is the same: $H_{i}=H,\;i=1,\ldots ,4$. In addition, we consider that all implants are perpendicular to the horizontal plane and are located symmetrically on the sides of the sagittal plane.

In modeling and analysis, we will use three coordinate systems.

**Figure 3:**
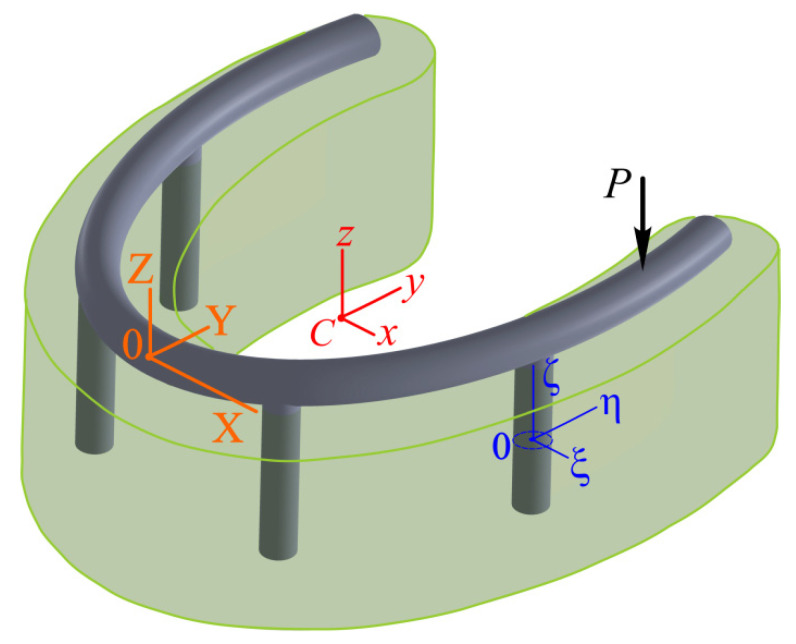
Calculation scheme of the force interaction of the elements of the bar structure as an assembly (implants–bar–overdenture).

**Figure 4:**
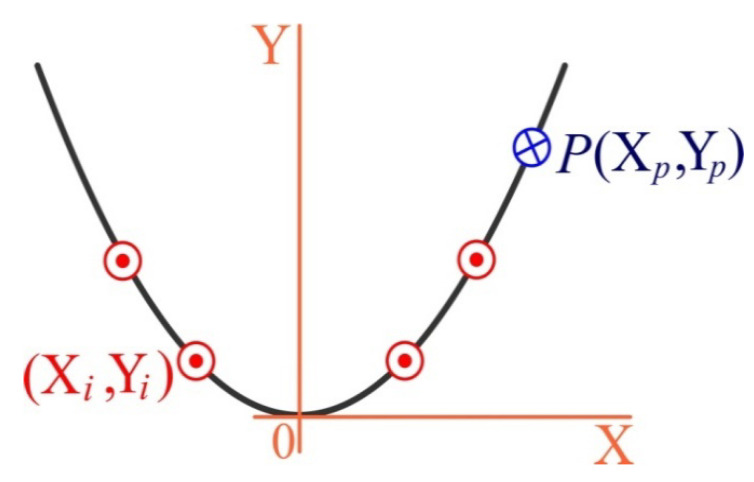
Original coordinate system (the OZ axis is directed at the reader): red—implants, blue—pole.

First of all, for the “terrain association” we select the original coordinate system OXYZ, with the beginning in the middle of the dentition, the upward axis Z, and the X,   Y axes in the distal and oral directions, respectively (Figure 3 and Figure 4).

Let us denote: $\left(X_{i},Y_{i}\right)$, i=1,…,4 as the implant coordinates in the original coordinate system; and $\left(X_{p},Y_{p}\right)$ as the coordinates of the application point (pole) of the mastication load, modeled by the vertical force P.

**Figure 5:**
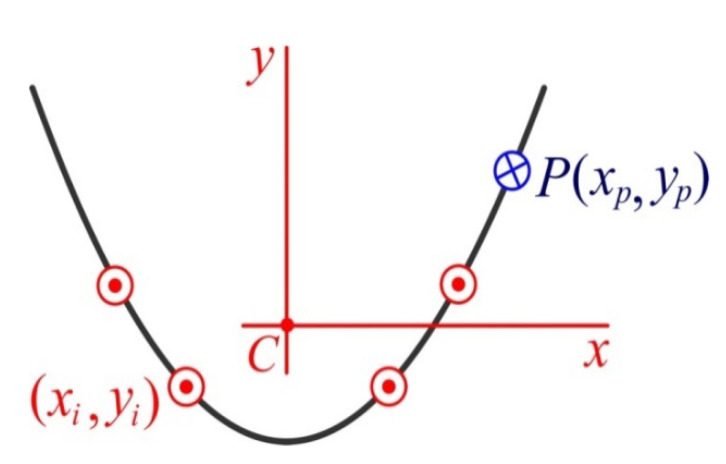
Central coordinate system (the Cz axis is directed at the reader): red—implants, blue—pole.

Therefore, the i-th implant occupies the segment $\left(X_{i},Y_{i},\;Z\right)$, Z∈[−H, 0]. In addition, we introduce the central coordinate system Cxyz (Figure 3 and Figure 5). To do this, we find the original coordinates of the center of mass (center of rigidity) of the four-implant system by Equation (1):

(1) $$X_{C}=\frac{1}{4}\sum_{i=1}^{4}X_{i},\;Y_{C}=\frac{1}{4}\sum_{i=1}^{4}Y_{i},\;Z_{C}=-\frac{1}{2}H.$$

From here, to reduce the record, we will omit the indexes in the sign of the sum, assuming that: $\sum_{i=1}^{4}t_{i}=\sum t_{i}$.

Thus, instead of the expressions in Equation (1), we have the following:

(2) $$X_{C}=\frac{1}{4}\sum X_{i},\;Y_{C}=\frac{1}{4}\sum Y_{i},\;Z_{C}=-\frac{1}{2}H.$$

As a result of the symmetry of the system with respect to the plane YZ, the central axes Cx, Cy, and Cz are parallel to the original axes, OX, OY, and OZ.

The transformation of the coordinate system occurs according to the following law:

(3) $$x=X-X_{C},\;y=Y-Y_{C},\;z=Z-Z_{C}.$$

Thus, z∈[−H/2, H/2]. Then, $x_{i}=X_{i}-X_{C}$, $y_{i}=Y_{i}-Y_{C}$ are the implant coordinates in the central coordinate system, and the corresponding coordinates of the pole are as follows:

(4) $$x_{p}=X_{p}-X_{C},\;y_{p}=Y_{p}-Y_{C}$$

Finally, to describe local phenomena in each implant, we introduce a local coordinate system $O_{i}\xi_{i}\eta_{i}\zeta_{i}$ (i=1,…,4) with the center in the middle of the implant and the axis $\zeta_{i}$ along its line. The $O_{i}\xi_{i}$ and $O_{i}\eta_{i}$ axes are parallel to the central axes.

With each coordinate system, we will associate three mutually perpendicular unit vectors, which we will call the base. In the original system, these will be vectors I, J,  K; in the central system, vectors i, j,  k; and in the local system, vectors $i_{i},\;j_{i},\;\;k_{i}$. Then an arbitrary vector, a, in a given coordinate system can be represented as a setup for base vectors. For example, for the central coordinate system:

(5) $$a=a_{x}i+a_{y}j+a_{z}k.$$

Values $a_{x}$, $a_{y}$, and $a_{z}$ are the coordinates of the vector in the base i, j, k.

Sometimes, instead of Equation (5), we will use the designation of a vector quantity in the form of a column or a transposed row, as follows: $a=\left(\begin{array}{l} a_{x}\\ a_{y}\\ a_{z} \end{array}\right)={\left(a_{x},a_{y},a_{z}\right)}^{T}$. 

When modeling, the operation of the vector product of two vectors will also be applied, denoted by a third-order determinant, as follows:

(6) $$a\times b=\left|\begin{array}{ccc} i & j & k\\ a_{x} & a_{y} & a_{z}\\ b_{x} & b_{y} & b_{z} \end{array}\right|=(a_{y}b_{z}-a_{z}b_{y})i+(a_{z}b_{x}-a_{x}b_{z})j+(a_{x}b_{y}-a_{y}b_{x})k=\;=\left(\begin{array}{l} a_{y}b_{z}-a_{z}b_{y}\\ a_{z}b_{x}-a_{x}b_{z}\\ a_{x}b_{y}-a_{y}b_{x} \end{array}\right).$$

#### 2.2.3. Key Equations of the Model

##### Kinematics

We will work in the central coordinate system. A rigid frame as the only absolutely rigid body in the general case has 6 degrees of freedom: 3 displacements—$\Delta_{x}$, $\Delta_{y}$, and $\Delta_{z}$—of the center *C* in the direction of the coordinate axes, and 3 rotations—$\Theta_{x}$, $\Theta_{y}$, and $\Theta_{z}$—around the coordinate axes. Thus, we are dealing with two vectors:

(7) $$\Delta =\Delta_{x}i+\Delta_{y}j+\Delta_{z}k,\;\Theta =\Theta_{x}i+\Theta_{y}j+\Theta_{z}k.$$

Then, the small displacement of any point of the rigid structure will be the superposition of the small displacement of the center and the small rotation around the center, as shown in Equation (8):

(8) u(x,y,z)=Δ+Θ×r,

where r(x,y,z)=x i+y j+z k is the radius vector of a point with coordinates (x,y,z). The symbol × denotes the vector product operation.

##### Statics

Let us construct vector equilibrium equations. Let $q_{i}=q_{xi}i+q_{yi}j+q_{zi}k$ be the vector of the force interaction of the i-th implant with a bone. P=−Pk is the vector of the vertical mastication load, $r_{i}=x_{i}i+y_{i}j+zk$ is a radius vector of the implant axis, and $r_{p}=x_{p}i+y_{p}j$ is a radius vector of the pole.

The equilibrium of the system will be ensured if the main vector and the main moment of all forces are equal to zero:

(9) $${\sum F}_{i}=\sum \int_{L}q_{i}dz+P=0,\;{\sum m_{0}(F}_{i})=\sum \int_{L}r_{i}\times q_{i}dz+r_{p}\times P=0.\;$$

Here, L=[−H/2, H/2] is the integration contour, which is the same for all implants; thus, the index i is omitted.

Two vector Equation (9) give 6 scalar equations in the coordinate notation, namely:

(10) $$\sum \int_{L}\left(\begin{array}{l} q_{xi}\\ q_{yi}\\ q_{zi} \end{array}\right)dz+\left(\begin{array}{l} 0\\ 0\\ -P \end{array}\right)=\left(\begin{array}{l} 0\\ 0\\ 0 \end{array}\right),\;\sum \int_{L}\left(\begin{array}{l} y_{i}q_{zi}-zq_{yi}\\ zq_{xi}-x_{i}q_{zi}\\ x_{i}q_{yi}-y_{i}q_{xi} \end{array}\right)dz+\left(\begin{array}{l} -y_{i}P\\ x_{i}P\\ 0 \end{array}\right)=\left(\begin{array}{l} 0\\ 0\\ 0 \end{array}\right).$$

##### Physical Relations

**Figure 6:**
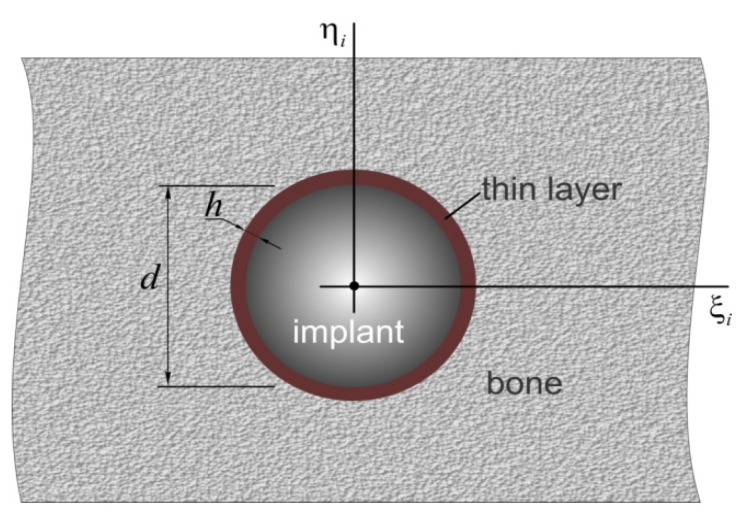
Scheme of the interaction of the implant with bone tissue: *h* is small layer thickness, *d* is the implant diameter.

Let us consider the issues of interaction between the implant and bone tissue in more detail. We propose that a rigid cylindrical implant is conjugated with the bone through a thin intermediate layer (Figure 6).

Such a calculation model is justified due to the fact that the implant material is much more rigid than the bone. The properties of the intermediate layer embody the properties of a thread in imperfect contact with fragments of regenerated young bone tissue. Under conditions of a small layer thickness h relative to the implant diameter d(h<<d), we take into account Winkler’s hypothesis, by which the contact normal $\sigma_{n}$ and tangential $\sigma_{\tau }$ stresses will be proportional to the transverse $u_{n}$ and vertical $u_{\tau }$ displacement of the implant relative to the bone [51], as shown in Equation (11):

(11) $$\sigma_{n}=-E_{b}\frac{u_{n}}{h},\;\sigma_{\tau }=-\frac{E_{b}}{2(1+\nu_{b})}\frac{u_{\tau }}{h},$$

where $E_{b}$ and $\nu_{b}$ are the height-averaged Young’s modulus and Poisson’s ratio of the bone, respectively.

Then, given that:

(12) $$q_{x}=\pi d\sigma_{x},\;q_{y}=\pi d\sigma_{y},\;q_{z}=\pi d\sigma_{\tau },$$

the physical relationships between contact forces and the displacements of implants can be represented as follows:

(13) $$q_{i}+Cu_{i}=0,\;i=1,\ldots ,4,\;$$

where $C=\left(\begin{array}{ccc} C_{n} & 0 & 0\\ 0 & C_{n} & 0\\ 0 & 0 & C_{\tau } \end{array}\right)$ is the rigidity matrix, and $C_{n}=E_{b}\frac{\pi d}{h}$ and $C_{\tau }=\frac{E_{b}}{2(1+\nu_{b})}\frac{\pi d}{h}$ are the bed rigidity coefficients [51].

##### Equation of Statics in Displacement

Bringing together Equations (8), (9), and (13), we obtain a system of two vector equations for two unknown vectors, Δ and Θ:

(14) $$-\sum \int_{L}C(\Delta +\Theta \times r_{i})dz+P=0,-\sum \int_{L}r_{i}\times C(\Delta +\Theta \times r_{i})dz+r_{p}\times P=0.$$

In the coordinate notation, the system in (14) will be a system of six scalar equations for finding six unknown values: $\Delta_{x}$, $\Delta_{y}$, $\Delta_{z}$ and $\Theta_{x}$, $\Theta_{y}$, $\Theta_{z}$.

##### Stress Calculation Scheme

By solving the system in Equation (14) using Equation (8), we can find the displacement of an implant:

(15) $$u_{i}=\Delta +\Theta \times r_{i},\;i=1,\ldots ,4;$$

Therefore, according to Equation (12), the linear forces in each layer are as follows:

(16) $$q_{i}=-C(\Delta +\Theta \times r_{i}),\;i=1,\ldots ,4;$$

Let us proceed to the consideration of the stress state of the implant. The integral force factors in the implant can be found from the differential equations of equilibrium of the rod as a system with distributed parameters, which is under tension to the vertical axis $\zeta_{i}$ and bilateral bending in the $\left(\xi_{i},\zeta_{i}\right)$ and $\left(\eta_{i},\zeta_{i}\right)$ planes. In vector form, this can be written as follows:

(17) $$\frac{dQ_{i}}{d\zeta }+q_{i}=0,\;\frac{dM_{i}}{d\zeta }+Q_{i}\times k=0,\;i=1,\ldots ,4,\;\zeta \in [-H/2,\;H/2].$$

Since the implants are parallel and have the same length, the index i in $\zeta_{i}$ is omitted. In Equation (17), $Q_{i}={\left(Q_{\xi \;i},Q_{\eta \;i},Q_{\zeta \;i}\right)}^{T}$ is the force vector, $Q_{\xi \;i}$ and $Q_{\eta \;i}$ are the transverse forces, $Q_{\zeta \;i}$ is the longitudinal force, $M_{i}={\left(M_{\xi \;i},M_{\eta \;i},M_{\zeta \;i}\right)}^{T}$ is the moment vector, $M_{\xi \;i}\;$ and $M_{\eta \;i}$ are the bending moments, and $M_{\zeta \;i}$ is the torque.

The system in Equation (17) should be solved under the initial conditions, which means that the recessed end of the implant is free from contact load, as shown below:

(18) $$Q_{\;i}(-H/2)=0,\;M_{\;i}(-H/2)=0,\;i=1,\ldots ,4.$$

From Equation (16), it can be seen that due to the linear dependence between $r_{i}$ and ζ, the vector functions $q_{i}$ also depend linearly on ζ; therefore, integration the system in (17) under the conditions of (18) does not cause fundamental difficulties.

Based on the integral characteristics found, one can find the stress in the implants as a linear function of the transverse coordinates. Normal axial stresses are calculated by the following formula:

(19) $$\sigma_{\zeta \;i}(\xi_{i},\eta_{i},\zeta )=\frac{Q_{\zeta i}(\zeta )}{S_{0}}+\frac{M_{\eta i}(\zeta )\xi_{i}}{J_{\eta i}}+\frac{M_{\xi i}(\zeta )\eta_{i}}{J_{\xi i}}.$$

Here, $S_{0}=\frac{\pi d^{2}}{4}$ is the cross-sectional area of the implant, and $J_{\xi i}=J_{\eta i}=\frac{\pi d^{4}}{64}$ are the moments of inertia of a circular cross section.

In particular, in the outer fibers of the implant, which are located in the plane of bending, the axial stresses can be written as follows:

(20) $$\sigma_{\zeta \;i}^{\pm }(\zeta )=\frac{Q_{\zeta i}}{S_{0}}\pm \frac{\sqrt{M_{\xi i}^{2}+M_{\eta i}^{2}}}{W},$$

where $W=\frac{J_{\xi i}}{d/2}=\frac{J_{\eta i}}{d/2}=\frac{\pi d^{3}}{32}$ is the moment of resistance of the cross section of the implant.

The stress in the bone in the vicinity of the implant is interpreted as stress in the intermediate Winkler layer. This can be easily found if the distribution along the length of the implant $q_{i}$ is known.

From Equation (13), the expressions for normal and tangential stresses in the bone are as follows:

(21) $$\sigma_{ni}(\zeta )=\frac{\sqrt{q_{\xi i}^{2}+q_{\eta i}^{2}}}{\pi d};\;\sigma_{\tau i}(\zeta )=\frac{q_{\zeta i}^{}}{\pi d}.$$

##### Strength Conditions

Based on the standard theories of strength used in the science of the strength of materials [67], expressions (20) and (21) can be used to estimate the load capacity of the “implant-bone” system under consideration. For implants subjected to compression with a bend, we will accept the first theory of strength, according to which the normal stresses with the highest modulus should not exceed the stress allowed for the implant material. For bone tissue (in fact, for the interface), we will use the fourth (energy) theory of strength, according to which the von Mises stresses in the intermediate layer should not exceed the value allowed for bone tissue. In symbolic notation, such conditions are written in terms of equivalent stresses:

(22) $$\underset{i}{\max}\;\underset{\zeta }{\max}\;\sigma_{eqi}^{impl}(\zeta )\leq {\left[\sigma \right]}^{impl},$$

(23) $$\underset{i}{\max}\;\underset{\zeta }{\max}\;\sigma_{eqi}^{bone}(\zeta )\leq {\left[\sigma \right]}^{bone}.$$

Here, $\sigma_{eqi}^{impl}(\zeta )\equiv \max\left\{\left|\sigma_{\zeta \;i}^{+}\right|,\left|\sigma_{\zeta \;i}^{-}\right|\right\}$ is the equivalent stress in the implant; $\sigma_{eqi}^{bone}(\zeta )\equiv \sqrt{\sigma_{n}^{2}+3\sigma_{\tau }^{2}}$ is the equivalent stress in the bone; and ${\left[\sigma \right]}^{impl}$ and ${\left[\sigma \right]}^{bone}$ are the allowable stress values for the implant and bone materials, respectively.

## 3. Results and Analysis

### 3.1. Analytical Solution

Note that as a result of the symmetrical arrangement of implants relative to the plane X=0, the introduced central axes will also be the principal axes of inertia. Under such conditions, the solution to the problem is greatly simplified. In particular, the system of vector equations of statics in displacements (14) in coordinate notation takes the following form:

(24) $$\left(\begin{array}{cccccc} C_{n}4H & 0 & 0 & 0 & 0 & 0\\ 0 & C_{n}4H & 0 & 0 & 0 & 0\\ 0 & 0 & C_{\tau }4H & 0 & 0 & 0\\ 0 & 0 & 0 & C_{\tau }a_{x}^{2}H & 0 & 0\\ 0 & 0 & 0 & 0 & C_{\tau }a_{y}^{2}H & 0\\ 0 & 0 & 0 & 0 & 0 & C_{n}a_{z}^{2}H \end{array}\right)\left(\begin{array}{c} \Delta_{x}\\ \Delta_{y}\\ \Delta_{z}\\ \Theta_{x}\\ \Theta_{y}\\ \Theta_{z} \end{array}\right)=\left(\begin{array}{c} 0\\ 0\\ -P\\ -Py_{p}\\ -Px_{p}\\ 0 \end{array}\right).$$

Here:

(25) $$a_{x}^{2}=\sum y_{i}^{2}+\gamma H^{2}/3,\;a_{y}^{2}=\sum x_{i}^{2}+\gamma H^{2}/3,\;a_{z}^{2}=\sum x_{i}^{2}+\sum y_{i}^{2},$$

(26) $$\gamma =\frac{C_{n}}{C_{\tau }}=2(1+\nu_{b}).$$

A very remarkable feature of the system of the linear algebraic Equation (24) is the diagonality of the matrix. This allows us to immediately write down the analytical solution, namely:

$$\Delta_{x}=0,\;\Delta_{y}=0,\;\Delta_{z}=-\frac{P}{4C_{\tau }H};$$

(27) $$\Theta_{x}=-\frac{Py_{p}}{C_{\tau }a_{x}^{2}H},\;\Theta_{y}=\frac{Px_{p}}{C_{\tau }a_{y}^{2}H},\;\Theta_{z}=0.$$

Note that due to the lack of horizontal components in the mastication load, there are no horizontal movements of the frame, and its rotation is around the vertical axis.

Thus, the displacements and rotations of the frame as a rigid whole are known. We used these results to search for implant displacements, force characteristics, and stresses.

Using Equation (8), we can express the vector of the displacements of the *i*-th implant through non-zero displacements and angles of rotation as follows:

(28) $$u_{i}(\zeta )=\left(\begin{array}{l} \Theta_{y}\zeta \\ -\Theta_{x}\zeta \\ \Delta_{z}+\Theta_{x}y_{i}-\Theta_{y}x_{i} \end{array}\right).$$

Taking into account the results of Equations (27) and (28), we finally obtain the followed:

(29) $$u_{i}(\zeta )=\frac{P}{C_{\tau }H}\left(\begin{array}{l} \frac{x_{p}\zeta }{a_{y}^{2}}\\ \;\frac{y_{p}\zeta }{a_{x}^{2}}\\ -\frac{1}{4}-\frac{y_{p}y_{i}}{a_{x}^{2}}-\frac{x_{p}x_{i}}{a_{y}^{2}} \end{array}\right).$$

By substituting the results of (28) and (29) into Equation (12), we can find the distribution of the loads on each of the implants as follows:

(30) $$q_{i}(\zeta )=\left(\begin{array}{l} -C_{n}\Theta_{y}\zeta \\ C_{n}\Theta_{x}\zeta \\ -C_{\tau }(\Delta_{z}+\Theta_{x}y_{i}-\Theta_{y}x_{i}) \end{array}\right)=\frac{P}{H}\left(\begin{array}{l} -\gamma \frac{x_{p}\zeta }{a_{y}^{2}}\\ -\gamma \;\frac{y_{p}\zeta }{a_{x}^{2}}\\ \frac{1}{4}+\frac{y_{p}y_{i}}{a_{x}^{2}}+\frac{x_{p}x_{i}}{a_{y}^{2}} \end{array}\right).$$

Let us write the vector differential equations in a coordinate-wise form as follows:

$$\frac{dQ_{\xi i}}{d\zeta }+q_{\xi i}=0,\;\frac{dQ_{\eta i}}{d\zeta }+q_{\eta i}=0,\;\frac{dQ_{\zeta i}}{d\zeta }+q_{\zeta i}=0;\;$$

(31) $$\frac{dM_{\xi i}}{d\zeta }+Q_{\eta i}=0,\;\frac{dM_{\eta i}}{d\zeta }-Q_{\xi i}=0,\;\frac{dM_{\zeta i}}{d\zeta }=0,\;z\in \left[-\frac{H}{2},\frac{H}{2}\right].$$

Taking Equation (30) into account, we can integrate these Equation (31) under the initial conditions (18). We obtain the following expressions for the vectors of the forces and moments:

(32) $$Q_{i}(\zeta )=\left(\begin{array}{l} Q_{\xi i}\\ Q_{\eta i}\\ Q_{\zeta i} \end{array}\right)=\left(\begin{array}{l} C_{n}\Theta_{y}\frac{1}{2}\left(\zeta^{2}-\frac{H^{2}}{4}\right)\\ -C_{n}\Theta_{x}\frac{1}{2}\left(\zeta^{2}-\frac{H^{2}}{4}\right)\\ C_{\tau }(\Delta_{z}+\Theta_{x}y_{i}-\Theta_{y}x_{i})\left(\zeta +\frac{H}{2}\right) \end{array}\right)=\frac{P}{H}\left(\begin{array}{l} \gamma \frac{x_{p}\zeta }{a_{y}^{2}}\frac{1}{2}\left(\zeta^{2}-\frac{H^{2}}{4}\right)\\ \gamma \;\frac{y_{p}\zeta }{a_{x}^{2}}\frac{1}{2}\left(\zeta^{2}-\frac{H^{2}}{4}\right)\\ -\left(\frac{1}{4}+\frac{y_{p}y_{i}}{a_{x}^{2}}+\frac{x_{p}x_{i}}{a_{y}^{2}}\right)\left(\zeta +\frac{H}{2}\right) \end{array}\right),$$

(33) $$M_{i}(\zeta )=\left(\begin{array}{l} M_{\xi i}\\ M_{\eta i}\\ M_{\zeta i} \end{array}\right)=\left(\begin{array}{l} \;C_{n}\Theta_{x}\frac{1}{6}{\left(\zeta +\frac{H}{2}\right)}^{2}\left(\zeta -H\right)\\ C_{n}\Theta_{y}\frac{1}{6}{\left(\zeta +\frac{H}{2}\right)}^{2}\left(\zeta -H\right)\\ 0 \end{array}\right)=\frac{P}{H}\left(\begin{array}{l} -\gamma \frac{x_{p}}{a_{y}^{2}}\frac{1}{6}{\left(\zeta +\frac{H}{2}\right)}^{2}\left(\zeta -H\right)\\ \gamma \;\frac{y_{p}}{a_{x}^{2}}\frac{1}{6}{\left(\zeta +\frac{H}{2}\right)}^{2}\left(\zeta -H\right)\\ 0 \end{array}\right).$$

Finally, according to the results of (30), (32), and (33) from Equation (20), we can find the distribution of the axial stresses in the outermost fibers of the i-th implant as follows:

(34) $$\sigma_{\zeta i}^{\pm }(\zeta )=-\frac{P}{S_{0}}\{\left(\frac{1}{4}+\frac{x_{p}x_{i}}{a_{y}^{2}}+\frac{y_{p}y_{i}}{a_{x}^{2}}\right)\left(\frac{\zeta }{H}+\frac{1}{2}\right)\pm \frac{4}{3}\gamma \frac{H}{d}\sqrt{{\left(\frac{Hx_{p}}{a_{y}^{2}}\right)}^{2}+{\left(\frac{Hy_{p}}{a_{x}^{2}}\right)}^{2}}{\left(\frac{\zeta }{H}+\frac{1}{2}\right)}^{2}\left(\frac{\zeta }{H}-1\right)\},$$

Based on Equation (21), the stress in the bone at the interface can be calculated as follows:

(35) $$\sigma_{ni}(\zeta )=\frac{P}{S_{l}}\gamma \sqrt{{\left(\frac{Hx_{p}}{a_{y}^{2}}\right)}^{2}+{\left(\frac{Hy_{p}}{a_{x}^{2}}\right)}^{2}}\frac{\zeta }{H},\;\sigma_{\tau i}(\zeta )=\frac{P}{S_{l}}\left(\frac{1}{4}+\frac{x_{p}x_{i}}{a_{y}^{2}}+\frac{y_{p}y_{i}}{a_{x}^{2}}\right).$$

Here, $S_{l}=\pi dH$ is the area of the lateral surface of the implant.

Finally, to calculate the equivalent stresses in the system, we obtain the following expressions:

(36) $$\sigma_{eqi}^{impl}(\zeta )=\left|\sigma_{\zeta i}^{\pm }(\zeta )\right|,\;$$

(37) $$\sigma_{eqi}^{bone}(\zeta )=\frac{P}{S_{l}}\sqrt{{\left(\frac{\gamma \zeta }{H}\right)}^{2}\left[{\left(\frac{Hx_{p}}{a_{y}^{2}}\right)}^{2}+{\left(\frac{Hy_{p}}{a_{x}^{2}}\right)}^{2}\right]+3{\left(\frac{1}{4}+\frac{x_{p}x_{i}}{a_{y}^{2}}+\frac{y_{p}y_{i}}{a_{x}^{2}}\right)}^{2}}.$$

From Equations (34), (36) and (37), it can be seen that the highest values of the equivalent stresses are achieved when ζ=H/2, i.e., at the upper end of the implant (in the area of the neck of the implant).

$$\underset{\zeta }{\max}\sigma_{eqi}^{impl}=\max\left\{\left|\sigma_{\zeta \;i}^{+}\left(\frac{H}{2}\right)\right|,\left|\sigma_{\zeta \;i}^{-}\left(\frac{H}{2}\right)\right|\right\},$$

(38) $$\sigma_{\zeta i}^{\pm }\left(\frac{H}{2}\right)=-\frac{P}{S_{0}}\left\{\frac{1}{4}+\frac{x_{p}x_{i}}{a_{y}^{2}}+\frac{y_{p}y_{i}}{a_{x}^{2}}\pm \frac{2}{3}\gamma \frac{H}{d}\sqrt{{\left(\frac{Hx_{p}}{a_{y}^{2}}\right)}^{2}+{\left(\frac{Hy_{p}}{a_{x}^{2}}\right)}^{2}}\right\}.$$

The same is true on the surface of the bone:

(39) $$\underset{\zeta }{\max}\sigma_{eqi}^{bone}=\frac{P}{S_{l}}\sqrt{{\frac{\gamma }{4}}^{2}\left[{\left(\frac{Hx_{p}}{a_{y}^{2}}\right)}^{2}+{\left(\frac{Hy_{p}}{a_{x}^{2}}\right)}^{2}\right]+3{\left(\frac{1}{4}+\frac{x_{p}x_{i}}{a_{y}^{2}}+\frac{y_{p}y_{i}}{a_{x}^{2}}\right)}^{2}}.$$

Note that the final expressions for stresses obviously do not contain the values of the bed coefficients. The results of the stresses depend only on the parameter γ, which characterizes the ratio of these coefficients (see Equation (26)).

### 3.2. Examples of Calculation

In this paragraph, we will give examples of calculating stresses in an overdenture with four implants with a length H=10 mm and a thickness d=4 mm, loaded with a vertical force P=100 N. In this case, $S_{0}=12.6\;\;{\mathrm{mm}}^{2}$ and $S_{l}=125.7\;{\mathrm{mm}}^{2}$. In addition, in the calculations we used $\nu_{b}=0.35$; therefore, γ=0.27 [9,68]. 

#### 3.2.1. Example 1

**Figure 7:**
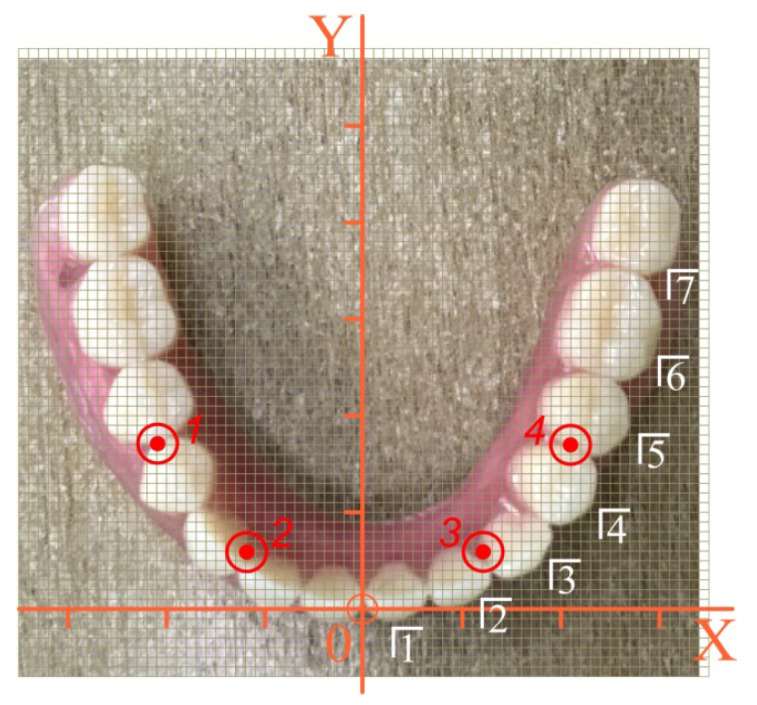
Numbering scheme and determination of the coordinates of the teeth and implants of a removable overdenture (the first variant of implant placement): red—implant number, white—tooth number.

Let us consider the configuration shown in Figure 7. The sequence of calculations is as follows.

We digitized the location of the implants in the original coordinate system: $X_{i}$ and $Y_{i}$ are the first two lines in Table 1. We agreed that the point of application of the force P (pole) coincided with the center of one of the teeth of the overdenture. We digitized the location of the pole in the original coordinate system: $X_{p}$ and $Y_{p}$ are the first two lines in Table 2.

According to Equation (1), we calculated the coordinates of the center *C*: $X_{C}=\;0\;\mathrm{mm}$, $Y_{C}=13.5\;\mathrm{mm}$, $Z_{C}=-5\;\mathrm{mm}$.

According to Equations (3) and (4), we listed the coordinates of implants and poles in the central coordinate system. The results are listed in the third and fourth lines of Table 1 and Table 2.

Having accepted the specified input data, the stress state of the implants and the nearby bone tissue was calculated at different positions of the mastication load according to Equations (34) and (35).

**Figure 8:**
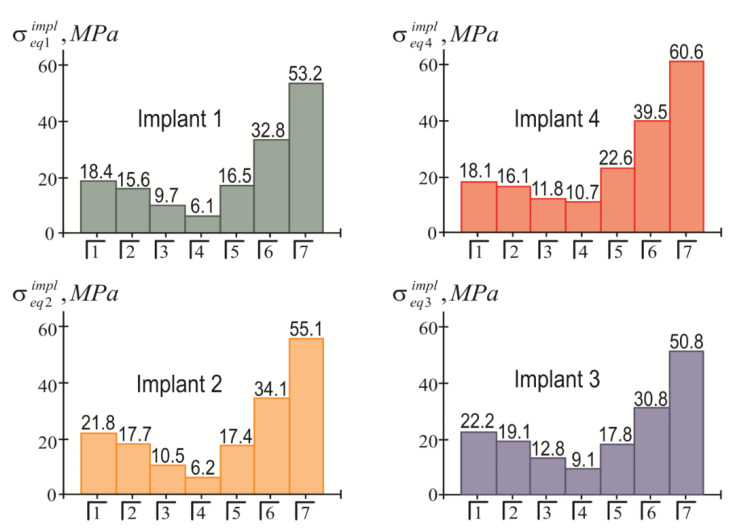
Dependence of the maximum equivalent stresses in implants on the location of the mastication load (the first variant of implant placement).

**Figure 9:**
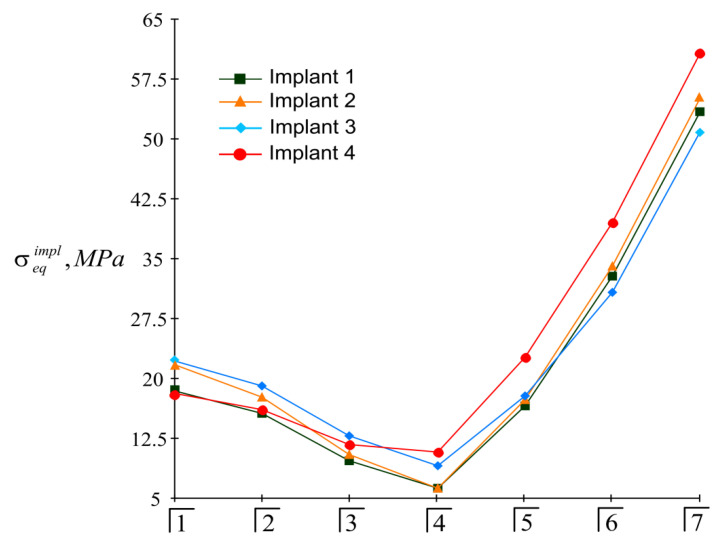
Comparative analysis of maximum equivalent stresses in implants at different mastication load locations (the first variant of implant placement).

The highest modulo values of the normal stresses in each implant were achieved at their upper end (in the region of the implant neck; ζ=H/2). Detailed information about the magnitude of the maximum equivalent stresses in the implants, depending on the location of the mastication load, is given in the diagrams shown in Figure 8. Finally, Figure 9 provides a summary of comparative information on the maximum equivalent stresses in the implants.

**Figure 10:**
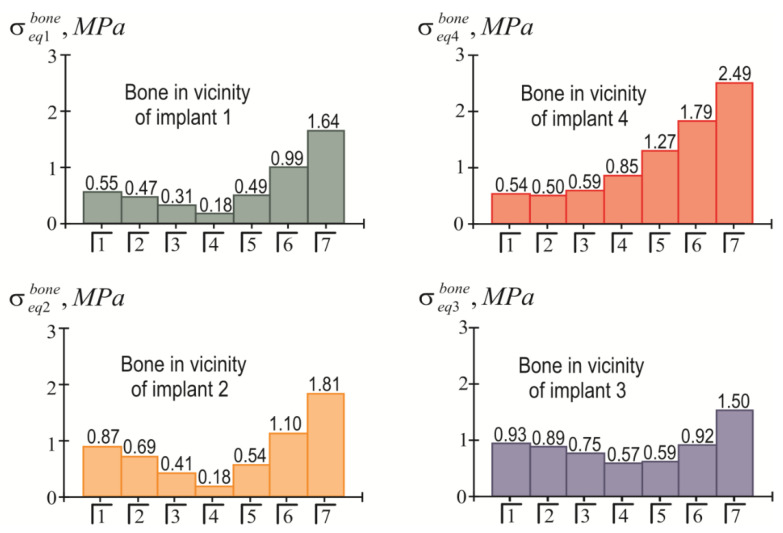
Dependence of maximum equivalent stresses in bone tissue on the location of the mastication load (the first variant of implant placement).

**Figure 11:**
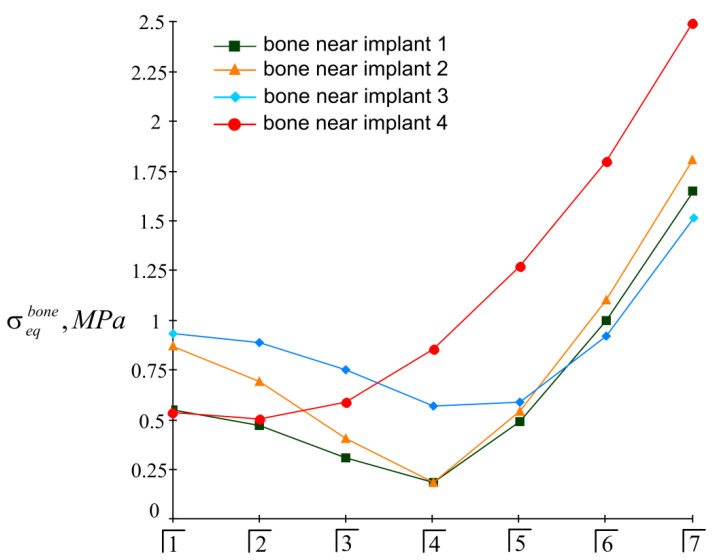
Comparative analysis of maximum equivalent stresses in bone tissue at different localizations of mastication load (the first variant of implant placement).

The distribution of equivalent stresses in the bone tissue adjacent to each of the implants indicates that the most problematic area for the bone is the near-surface zone ζ=H/2. The effect of the position of the load pole on the value of the highest equivalent stress in the bone near each implant is shown in Figure 10. In turn, Figure 11 allows us to compare the levels of the stresses near different implants.

Taking into account that ${\left[\sigma \right]}^{impl}=150\;\mathrm{MPa}$, based on the information presented in Figure 8 and Figure 9, we affirmed that the holding capacity of the metal frames at a given load level was provided with a significant margin of safety. The life cycle of an “overdenture-jaw” structure is determined by the holding capacity of the bone tissue adjacent to the implants. Here, as can be seen from Figure 10 and Figure 11, this depends on the location of the load application. The bone feels least comfortable in the vicinity of the fourth (supporting) implant, especially when biting with teeth 5, 6, and 7. This trend is explained by the lever effect (the removal of the load pole from the supporting fourth implant). However, even in this situation, if we accept that ${\left[\sigma \right]}^{bone}=3\;\mathrm{MPa}$, it can be argued that the strength of the jaw under load (P=100 N) is provided regardless of the location of the mastication load, because the strength condition of Equation (23) is always met (even when loading the seventh tooth). In other words, we have the right to design a full-fledged overdenture containing 14 teeth.

In the case of a weak bone with a smaller ${\left[\sigma \right]}^{bone}$ value, it is necessary either to reduce the mastication load or to abandon the load of the seventh tooth (or even tooth number 6), for example, by shortening the cantilever.

#### 3.2.2. Example 2

**Figure 12:**
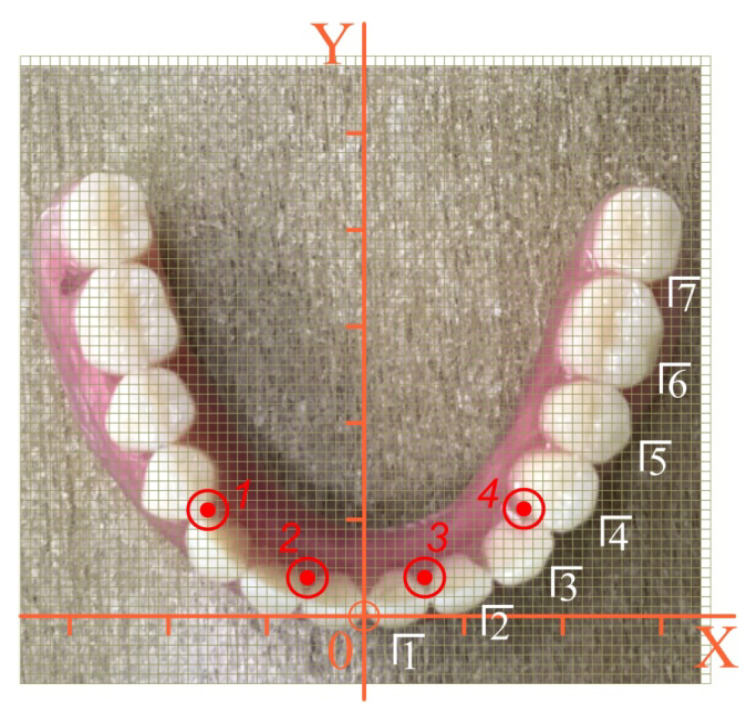
Numbering scheme and determination of the coordinates of the teeth of a removable overdenture (the second variant of implant placement): red—implant number, white—tooth number.

Let us now consider the configuration of a four-implant overdenture, shown in Figure 12. As in the previous example, the implants were located at the vertices of an equilateral trapezoid; however, the trapezoid is smaller. 

The coordinates of the implants in the original and central coordinate systems are included in Table 3. The pole coordinates are still presented in Table 2.

**Figure 13:**
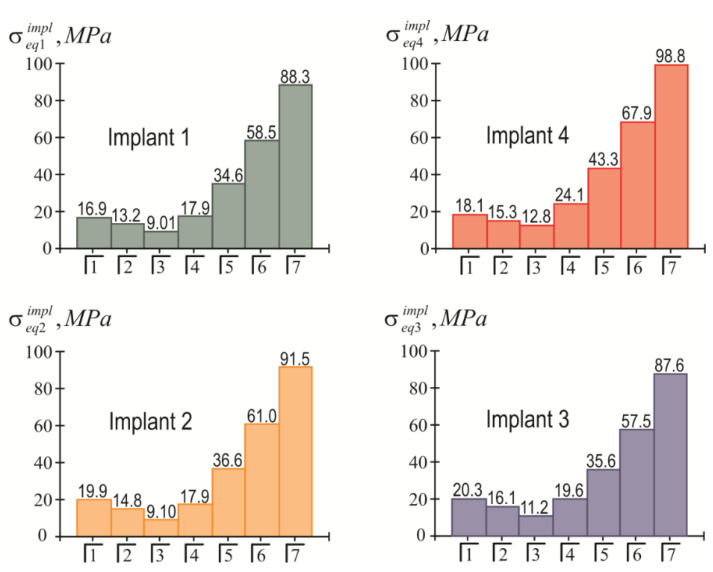
Dependence of maximum equivalent stresses in implants on the localization of the mastication load (the second variant of implant placement).

**Figure 14:**
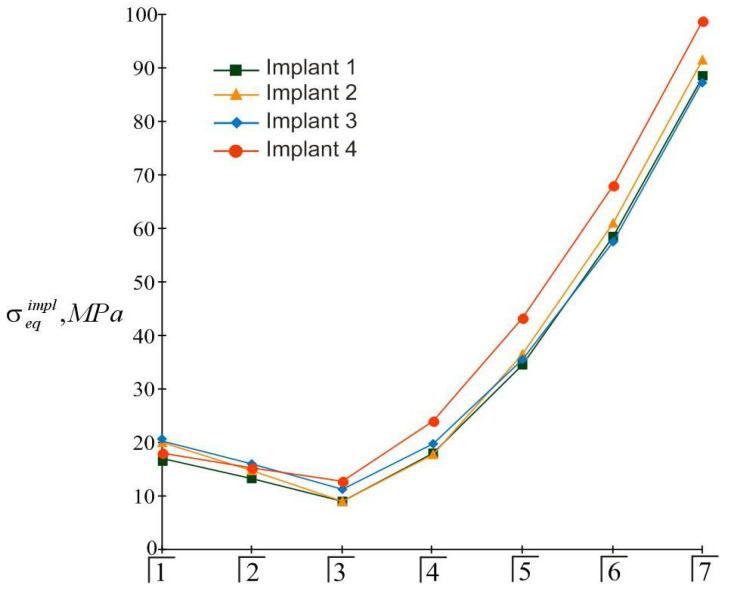
Comparative analysis of maximum equivalent stresses in implants at different localizations of the mastication load (the second variant of implant placement).

**Figure 15:**
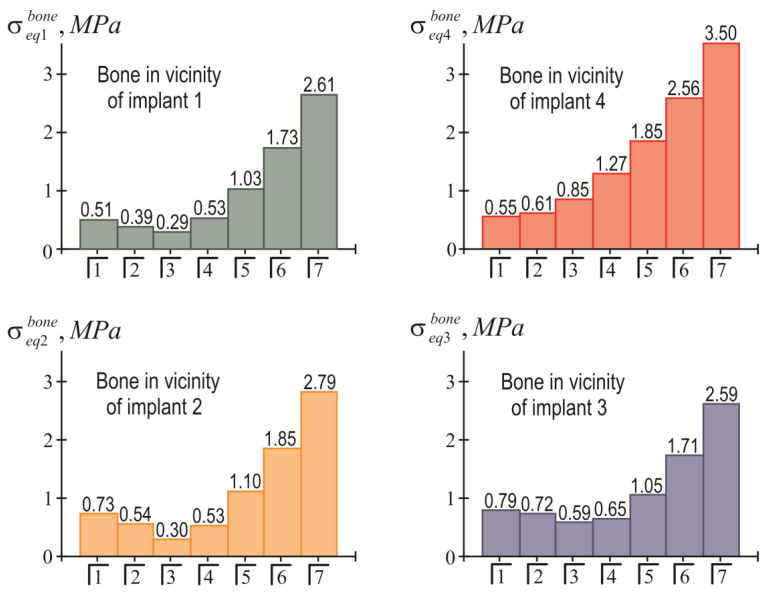
Dependence of maximum equivalent stresses in bone tissue on the location of the mastication load (the second variant of implant placement).

**Figure 16:**
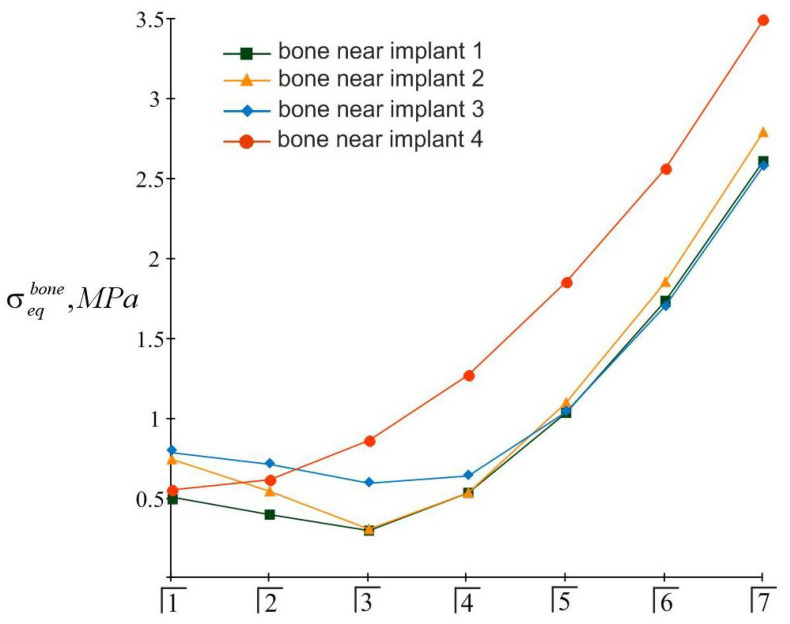
Comparative analysis of maximum equivalent stresses in bone tissue at different localizations of the mastication load (the second variant of implant placement).

The results of the calculations of the highest equivalent stresses in the implants and bone tissue are presented in Figure 13, Figure 14, Figure 15 and Figure 16.

Based on the analysis of this graphic material, the following should be noted: As before, the inequality in Equation (22) is satisfied for all implants, i.e., the integrity of the implants is ensured regardless of the place of application of the mastication load. At the same time, the equivalent stress in the bone near the fourth implant when the seventh tooth is loaded may exceed the allowable stress level: $3.5\;\mathrm{MPa}>{\left[\sigma \right]}^{bone}=3\;\mathrm{MPa}$.

This is a manifestation of the lever effect, which increases with a decrease in the size of the trapezium at the implant’s location. In such a situation, it is recommended to either limit the mastication load, taking, for example, P=80 N, or to allow the overdenture to be loaded no further than the sixth tooth; that is, to shorten the prosthetic dentition.

## 4. Discussion

The rapid development of computer technology and computational methods for solving problems has not bypassed the sphere of biomechanics and bioengineering [69]. Recently, digital technologies have significantly changed the clinical practice of diagnosis, orthopedic planning, surgery, and implant-supported restoration. With the advent of specialized software for clinicians and dental technicians, it has become possible to visualize final results and improve the interaction between the doctor and the patient, as well as the cooperation between the orthopedic dentist, surgeon, and dental technician, achieving a better quality final result.

An approach to the analysis of dental problems based on the use of advanced simulation packages is increasingly used in modern orthopedics. This approach usually includes the following steps [70,71,72,73]:

− A computed tomography (CT) analysis of the state of the jaw and dentition;
− The identification of the mechanical properties of bone tissue and teeth according to the established CT density distribution;
− The development of a solution to the problem of jaw interaction with an overdenture variant based on finite element analysis using ANSYS, SolidWorks, or ABACUS software systems;
− Overdenture design based on the results of the numerical analysis.

This approach provides sufficiently comprehensive and detailed information on the spatial distribution of the stress–strain state in heterogeneous bone tissue, teeth, and the overdenture; however, it is quite expensive and is usually associated with additional biological losses for patients [74]. First, it requires certified software products, certified hardware, and certified users. Secondly, and most importantly, obtaining detailed and reliable initial information is always associated with the need to specify a large amount of sufficiently accurate input data regarding the spatial configuration and physical and mechanical properties of the objects under study. Moreover, the more accurate the mathematical model, the more verified input information it needs. As a result, the research process is overgrown with unnecessary details, since not all factors affect the final result in the same way. The art of modeling consists of building a model that operates on the smallest possible set of input data and at the same time provides adequate estimates of the most significant output parameters.

This approach was used in our study to build a mechanical and mathematical model of the interaction of a four implant-supported overdenture with the mandible.

The distribution of the loads and stresses in dental structures attached to the jaw with the help of implants has been studied in many works, and general approaches to such problems can be divided into three global areas. The first direction is based on analytical methods [75,76,77,78,79], the works of the second direction are based on the finite element method and the setting of a virtual experiment [80,81,82,83,84], and the third direction uses the means of model and field experiments [85,86,87,88,89]. Our article develops the first analytical line of research.

Bibliosemantic and content analyses have shown that currently, the analytical direction is the least developed and is the least represented in the research literature. In particular, the analytical prediction of the forces and moments that occur on dental implants, as well as the problems surrounding the safe loading of the overdenture, are considered in [75]. In another study [76], an analytical model of the interaction of a single implant with bone tissue was used for the further calculation of fatigue strength. Another article [77] proposed a simple mathematical model for determining the rigidity of the dental implant–bone system. The work in [78] considers the classical problem of the theory of elasticity with regard to the contact interaction between the implant and the jawbone and studies the stress–strain state of the cancellous jawbone near a single implant under an occlusal load. An analytical determination of the optimal torque for the installation of a screw dental implant was carried out in [79]. At the same time, analytical studies of the dependences of equivalent stresses in the ensemble of implants and adjacent bone tissue on the coordinates of the location of the implants and on the localization of the mastication load have not been carried out. Our approach helps to overcome this theoretical gap and, in this sense, stakes a claim to novelty.

The simplicity and transparency of the analytical model, which allows one to quickly assess the stress and limit state of the prosthetic structure, is certainly very attractive. However, another important point that necessarily accompanies each study is the question of the adequacy of the model and the reliability of the results. In order to compare our analytical results with computer FE simulation data, among many well-known publications, we selected articles [90,91], in which the layout of implants, as well as the magnitude and localization of the mastication load, are most similar to our examples. This is how the authors of [90] considered the transfer of a vertical load of 100 N from a four-implant overdenture to the jawbone. Three types of loads were applied: at the central incisor area, at the premolar region, and at the molar area. According to Table 3 and Figure 8 in [90], in the case of loading the incisors, the peak values of the von Mises stresses in the implants were approximately 70 MPa, and in the trabecular bone were 2–3 MPa. Under the action of a load in the molar region, the corresponding stress values for the implants were 80–100 MPa, and for bone were 2–3 MPa. Our results (Figure 13, Figure 14, Figure 15 and Figure 16) indicate that when the overdenture was loaded in the area of the first four teeth, the highest equivalent stresses for the implants were in the range of 10–20 MPa, and, for bone, 0.5–1 MPa. The load of the overdenture in the area of the sixth tooth gave a value of 67.9 MPa for the implant and 2.56 MPa for the bone. Thus, we can appreciate the coincidence of the orders of the results of analytical and computer simulations, as well as the correct display of stress growth trends when the location of the pole changes in the distal direction. We explain some quantitative differences by the fact that our model did not take into account the stress concentration in the hollows of thread. Similar results are obtained by comparing our results acquired at a load of 100 N with the half-sum of the stress values under vertical forces of 60 N and 130 N, obtained by numerical analysis in [91].

## 5. Conclusions

In this article, a mechanical and mathematical model of the interaction of a completely removable bar-retaining four implant-supported overdenture system with the bone tissue of the mandibula was developed. This model makes it possible to analytically assess the stress level of implants and nearby bone tissue under a given mastication load.

The key hypotheses of the model are the interpretation of the “implants–bar–overdenture” system as an absolutely rigid spatial frame and the description of the mechanical properties of the “implant–bone” interface using Winkler’s hypothesis. In addition, bone resorption is not taken into account.

The model operates with a minimum set of input parameters that must be specified: implant size, implant location coordinates at the vertices of an equilateral trapezoid, overdenture tooth coordinates to which the mastication load will be applied, Poisson’s ratio and the allowable tension for the bone tissue, the amount of mastication load, and the desired length of the dentition.

For the first time, the analytical dependences of equivalent stresses in implants (38) and nearby bone tissue (39) on the coordinates of the location of the implants and the coordinates of the application of the mastication load were obtained. These expressions, with a fixed geometry of the implant location, allow one to find the allowable mastication load for a given length of the dentition of the overdenture or to set the dimensions of the overdenture (the maximum number of loaded teeth) for a given mastication load.

Two examples of calculations with greater and lesser distance between the anterior and posterior implants were considered here. It was established that in both cases, under real mastication loads, the integrity of the titanium implants was ensured.

In the case of a large distance between the implants for “healthy” bone with a medium allowable stress ${\left[\sigma \right]}^{bone}=3\;\mathrm{MPa}$, a load P=100 N is safe if applied to any tooth (from the first to the seventh). This gives us permission to use a full-length overdenture.

With a small distance between the anterior and posterior implants, when the connecting bar is almost straight, a load of 100 N, applied to the seventh tooth, can be problematic and can disrupt the integrity of the bone near the supporting (fourth) implant.

The comparison of our analytical results with numerical analysis data from the literature confirms the ability of the analytical approach to correctly describe the qualitative patterns of the behavior of a multi-support overdenture under load.

With the help of the developed technique, it is also possible to estimate the influence of implant sizes on the stress state of the “overdenture–bone” system. In addition, the arbitrary location of the implants (at the vertices of an irregular quadrangle), their different sizes and orientations, and the inhomogeneity of the bone at depth can be taken into account.

## Figures and Tables

**Table 1:** Coordinates of implants in the original and central coordinate system (the first variant of implant placement).

Implant No.	1	2	3	4
$X_{i},\;\mathrm{mm}$	−21	−12	12	21
$Y_{i},\;\mathrm{mm}$	17	6	6	17
$x_{i},\;\mathrm{mm}$	−21	−12	12	21
$y_{i},\;\mathrm{mm}$	5.5	−5.5	−5.5	5.5

**Table 2:** Coordinates of the pole (tooth) in the original and central coordinate system.

Tooth No.	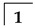	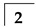	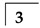	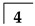	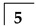	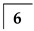	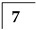
$X_{p},\;\mathrm{mm}$	3	9	15	19	23	25	28
$Y_{p},\;\mathrm{mm}$	1	3	7	13	20	29	40
$x_{p},\;\mathrm{mm}$	3	9	15	19	23	25	28
$y_{p},\;\mathrm{mm}$	−10.5	−8.5	−4.5	1.5	8.5	17.5	28.5

**Table 3:** Implant coordinates in the original and central coordinate system (the second variant of implant placement).

No. of Implant	1	2	3	4
$X_{i},\;\mathrm{mm}$	−16	−6	6	16
$Y_{i},\;\mathrm{mm}$	11	4	4	11
$x_{i},\;\mathrm{mm}$	−16	−6	6	16
$y_{i},\;\mathrm{mm}$	3.5	−3.5	–3.5	3.5

## Data Availability

Data are contained within the article.
